# Emerging role of extracellular vesicles and exogenous stimuli in molecular mechanisms of peripheral nerve regeneration

**DOI:** 10.3389/fncel.2024.1368630

**Published:** 2024-03-15

**Authors:** Yara Izhiman, Leyla Esfandiari

**Affiliations:** ^1^Esfandiari Laboratory, Department of Biomedical Engineering, College of Engineering and Applied Sciences, University of Cincinnati, Cincinnati, OH, United States; ^2^Department of Environmental and Public Health Sciences, College of Medicine, University of Cincinnati, Cincinnati, OH, United States; ^3^Department of Electrical and Computer Engineering, College of Engineering and Applied Sciences, University of Cincinnati, Cincinnati, OH, United States

**Keywords:** peripheral nerve regeneration, extracellular vesicles, electrical stimulation, mechanotherapy, signaling pathways

## Abstract

Peripheral nerve injuries lead to significant morbidity and adversely affect quality of life. The peripheral nervous system harbors the unique trait of autonomous regeneration; however, achieving successful regeneration remains uncertain. Research continues to augment and expedite successful peripheral nerve recovery, offering promising strategies for promoting peripheral nerve regeneration (PNR). These include leveraging extracellular vesicle (EV) communication and harnessing cellular activation through electrical and mechanical stimulation. Small extracellular vesicles (sEVs), 30–150 nm in diameter, play a pivotal role in regulating intercellular communication within the regenerative cascade, specifically among nerve cells, Schwann cells, macrophages, and fibroblasts. Furthermore, the utilization of exogenous stimuli, including electrical stimulation (ES), ultrasound stimulation (US), and extracorporeal shock wave therapy (ESWT), offers remarkable advantages in accelerating and augmenting PNR. Moreover, the application of mechanical and electrical stimuli can potentially affect the biogenesis and secretion of sEVs, consequently leading to potential improvements in PNR. In this review article, we comprehensively delve into the intricacies of cell-to-cell communication facilitated by sEVs and the key regulatory signaling pathways governing PNR. Additionally, we investigated the broad-ranging impacts of ES, US, and ESWT on PNR.

## Graphical abstract

Extracellular vesicles, along with exogenous stimuli, play a pivotal role in mediating intercellular communication and augmenting peripheral nerve regeneration, offering promising clinical applications.

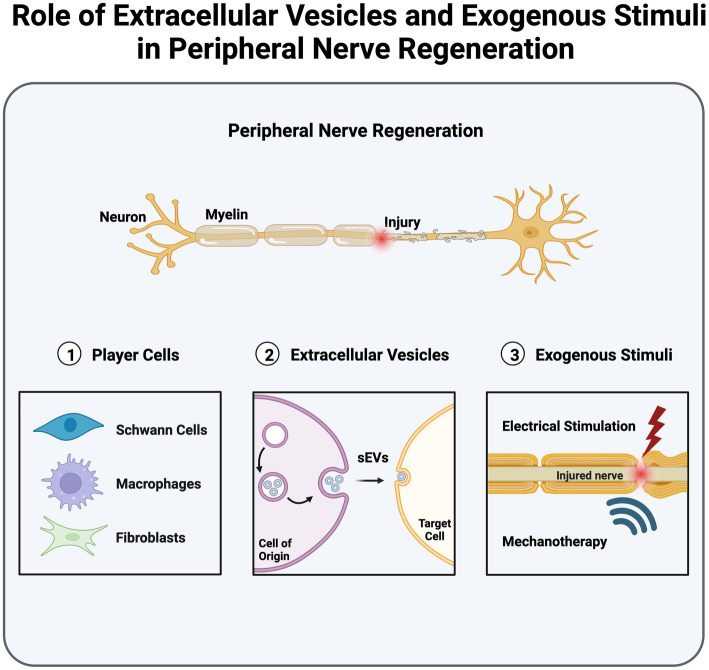

## 1 Introduction

Peripheral nerve injury (PNI) is a prevalent clinical concern, presenting in approximately 5% of patients with trauma associated with nerve root and brachial plexus injuries (Noble et al., 1998; Robinson, 2022). Although the peripheral nervous system (PNS) is capable of autonomous healing through peripheral nerve regeneration (PNR), the likelihood of complete nerve regrowth following an injury depends, in part, on the injury grade and gap size between the proximal and distal ends of the injured nerve (Lundborg et al., 1982; Menorca et al., 2013). Unfortunately, the achievement of successful nerve regeneration and subsequent functional recovery are not always assured. This inherent uncertainty underscores the pressing need to advance current therapeutic approaches aiming to accelerate and augment PNR. To this end, a meticulous exploration of the participating intercellular pathways is hypothesized to identify the pivotal molecular and cellular mechanisms that can potentially enhance regeneration.

PNR constitutes a multistep process driven by a cascade of intercellular communications that occurs in three stages, namely, Wallerian degeneration, axonal elongation, and nerve remyelination. The process of Wallerian degeneration is responsible for clearing cellular and myelin debris following a nerve injury, thus establishing a clear pathway for axonal regeneration within the peripheral nerve microenvironment. Axonal regeneration is determined by the formation of a growth cone at the proximal end of an injured nerve, which drives the endogenous regeneration and outgrowth of an axon. The pace of axonal elongation is dictated by the nature of an injury; for instance, a crush injury prompts a more rapid rate of elongation, approximately 3–4 mm/day. In contrast, a transection injury leads to a slower rate of elongation, averaging approximately 2.5 mm/day (Romero-Ortega, 2014; Jobe et al., 2020). The timeline of the axonal elongation process varies, spanning from weeks to months, depending upon the severity and nature of injury. In the final stage of regeneration, remyelination concludes with the establishment of properly regenerated nerve fibers and reinnervation of the target tissue.

Recent studies have revealed the important role of small extracellular vesicles (sEVs) in intercellular communication, which is crucial for PNR. The sEVs associated with the PNS actively participate in the regenerative cascade by facilitating communication through their molecular cargos, including proteins, lipids, and micro-RNAs (miRNAs), among cells (Hercher et al., 2022). Although not yet fully elucidated, this mediation is indispensable in cellular communication during regeneration, most notably among nerve cells, Schwann cells (SCs), macrophages, and fibroblasts. Moreover, non-invasive therapeutic treatments, such as electrical stimulation (ES), ultrasound (US), and extracorporeal shock wave therapy (ESWT), are reported to enhance PNR along with sEV secretion. Notably, ES treatment has been observed to enhance axonal growth through increased secretion and uptake of sEVs (Hu et al., 2019). Conversely, mechanotherapies like US and ESWT are associated with enhanced and altered sEVs secretion (Zeng et al., 2019; Gollmann-Tepekoylu et al., 2020; Ye et al., 2023). Further investigation into this phenomenon could provide deeper insights into the mechanisms driving associated regeneration.

Several review articles focus on the molecular and physiological aspects of peripheral nerve regeneration, as well as the state-of-the-art PNI treatment (Webber and Zochodne, 2010; Jessen and Mirsky, 2019; Hussain et al., 2020; Li et al., 2020; Nagappan et al., 2020; Zuo et al., 2020; Gurung et al., 2021) and, the role of extracellular vesicles (EVs) in PNR (Ching and Kingham, 2015; Budnik et al., 2016; Qing et al., 2018; Bischoff et al., 2022; Hercher et al., 2022). However, certain knowledge gaps still exist concerning the impact of exogenous stimuli on molecular mechanisms and sEV secretion during PNR. In this review, we highlight the vital molecular mechanisms governing PNR. We also provide a comprehensive review of the role of sEVs in intercellular communication within the regenerative cascade, along with the notable therapeutic potential of sEVs in enhancing PNR. Finally, we delve into the potential of external stimuli, namely ES, US and ESWT, on sEV secretion and associated molecular mechanisms during PNR. The aim of this review is to shed light on possible future directions that may address the challenges associated with peripheral nerve repair.

## 2 Player cells and intracellular signaling pathways in peripheral nerve regeneration

The PNS consists of three distinct cell types: neuronal, stromal, and glial cells. Neuronal cells consist of efferent (motor) neurons that directly receive signals from the central nervous system (CNS), as well as sensory neurons, such as the dorsal root ganglion (DRG), which translate signals into fine sensations (Jobe et al., 2020). Stromal cells, such as fibroblasts, constitute the non-neural connective tissue in nerves (Menorca et al., 2013). Fibroblasts generate proteins and extracellular matrix (ECM) components, such as collagen (I and II) and laminin, which provide structural support and protection to nerves. Within the PNS, glial cells are categorized into three main types: enteric cells, satellite cells, and SCs. Enteric cells maintain sensory homeostasis in the gastrointestinal tract. In contrast, satellite cells provide exclusive nutritional and structural support to neurons within the PNS. Satellite cells aid axonal elongation after peripheral nerve injury by enhancing proliferation, regeneration-associated gene expression, and macrophage recruitment (Krishnan et al., 2018; Avraham et al., 2020). Avraham et al. (2020) identified the regulatory role of satellite cells in the activation of peroxisome proliferator-activated receptor-α (PPARα) signaling in nerve cells to promote axonal regeneration. However, understanding the biology of satellite cells remains an ongoing challenge, resulting in a limited knowledge of their molecular role in PNR (Krishnan et al., 2018; Jager et al., 2020). SCs play a vital role in nerve development, myelination, and protection. They align themselves along the axons, enveloping them in a protective myelin sheath that improves conduction velocity, reduces axonal membrane capacitance, and increases axonal resistance to ion flux across the plasma membrane (Menorca et al., 2013; Rasband, 2016). Additionally, they contribute to axonal regrowth by forming longitudinally aligned tubular structures called Bands of Büngner, which provide structural support and guidance to regrowing axons (Panzer et al., 2020). Besides these resident cells, the PNS comprises immune cells and macrophages that are actively involved in nerve protection and pain modulation (Gaudet et al., 2011; Defrancesco-Lisowitz et al., 2015).

**Figure 1:**
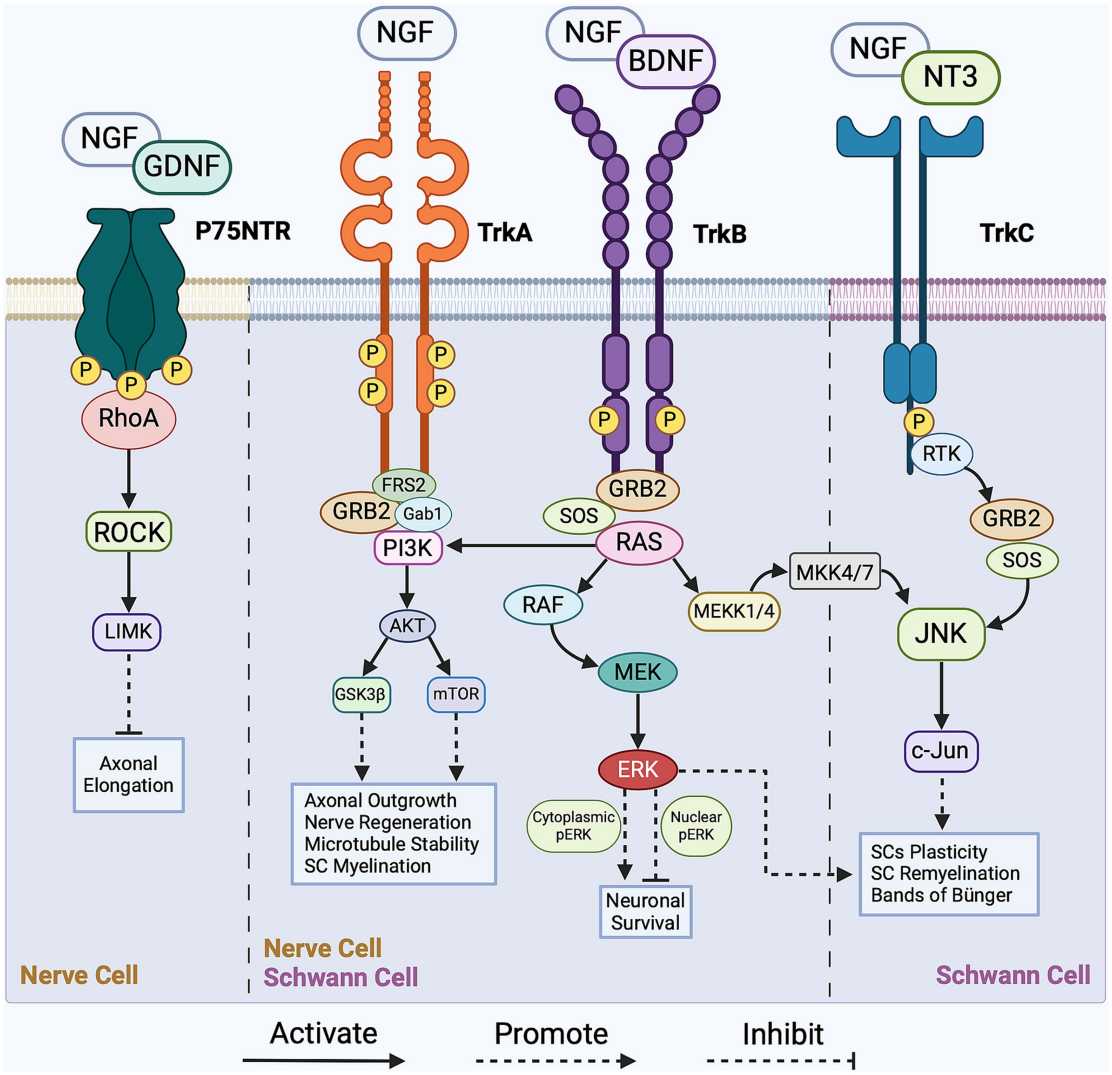
Overview of the pivotal signaling cascades in peripheral nerve regeneration (PNR). The intracellular regulation of Schwann cells (SCs) is facilitated by the JNK/c-Jun, PI3K/Akt/mTOR, and RAS/ERK signaling pathways with main activators being growth factors BDNF, NGF, and NT3. As for nerve cells, intracellular regulation is facilitated by the RhoA/ROCK, PI3K/Akt/mTOR, and RAS/ERK signaling pathways with main activators being growth factors NGF, GDNF, and BDNF. These intracellular pathways regulate axonal outgrowth, SCs reprogramming, nerve myelination and survival. For a more detailed schematic, please refer to Li et al. (2020). NGF, nerve growth factor; BDNF, brain-derived neurotrophic factor; GDNF, glial cell line-derived neurotrophic factor; NT3, neurotrophin-3; Trk, tropomyosin receptor kinase A; p75 NTR, p75 neurotrophin receptor; FRS-2, fibroblast growth factor (FGF) receptor substrate 2; GRB-2, growth factor receptor-bound protein 2; SOS, son of sevenless; Gab1, GRB2-associated binding protein 1; MEK, mitogen-activated protein kinase kinase; MEKK4/7, mitogen-activated protein kinase kinase kinase 4/7; MKK4/7, mitogen-activated protein kinase kinase 4/7; ERK, extracellular signal-regulated kinase; RTK, receptor tyrosine kinase; c-Jun, Jun proto-oncogene; JNK, c-Jun N-terminal kinase; ROCK, Rho-associated protein kinase; LIMK, LIM domain-containing kinase; mTOR, mammalian target of rapamycin; GSK3β, glycogen synthase kinase-3β; RhoA, Ras homolog family member A; PI3K, phosphoinositide 3-kinase; AKT, Protein kinase B; RAS, rat sarcoma; RAF, rapidly accelerated fibrosarcoma.

Communication among cells within the regenerative cascade plays a crucial role in intracellular signaling, which, in turn, regulates myelin clearance, SC reprogramming, axonal outgrowth, and nerve remyelination. Notable intracellular signaling pathways that orchestrate regeneration are activated by mitogen-activated protein kinases (MAPKs), tyrosine kinase receptors (Trk), non-receptor tyrosine kinase (NRTK), cytokine receptors, and p75 neurotrophin receptor (p75NTR). The MAPK family comprises three subfamilies of protein kinases: extracellular signal-regulated kinase (ERK), stress-activated Jun proto-oncogene (c-Jun) N-terminal kinase (JNK), and stress-activated p38 kinase. Following nerve injury, the accumulation of MAPK activates both the JNK/c-Jun and ERK pathways in nerve cells, concurrently sealing the injured axonal membrane and initiating Wallerian degeneration (Agthong et al., 2006; Gao et al., 2013). Given the complexity of the regenerative process, the intracellular signaling pathways establish an interrelationship among various cell types to facilitate nerve regrowth. In this section, we highlight the prominent regulatory signaling pathways governing each stage of the regenerative cascade. Focusing on the regulatory role of the p75 neurotrophin and Trk receptors, Figure 1 presents a simplified overview of the key signaling pathways operating within nerve cells and SCs during PNR.

### 2.1 Schwann cell reprogramming

Following peripheral nerve injury, elevated calcium levels surge through both the axoplasm and surrounding SCs. This initiates an elongated depolarization wave along the axonal membrane, inducing the upregulation of injury-related genes in the nerve cells. The calcium influx triggers calcium-dependent ion channels to initiate axonal membrane sealing in preparation for growth cone formation (Ziv and Spira, 1995, 1997; Chierzi et al., 2005; Bradke et al., 2012; Nagappan et al., 2020; Rigoni and Negro, 2020). Subsequently, within the first 48 h, SCs undergo Wallerian degeneration, adopting a non-myelinating phenotype (reprogramming). This reprogramming involves the downregulation of myelin-associated factors, including transcription factor early growth response protein-2 (EGR2), also known as Krox20, cholesterol synthesis enzymes, structural protein zero (P0), myelin basic protein (MBP), and membrane-associated glycoproteins (MAG; Chen et al., 2007; Jessen and Mirsky, 2008, 2016). In addition, non-myelinating SCs migrate from the distal nerve stump to the area of injury, which are reported to assist in myelin clearance and guide axonal elongation through growth factor secretion (Chen et al., 2007; Jessen and Mirsky, 2019; Min et al., 2021).

One key effector in the modulation of the myelinating state of SCs is the inhibition of the GTPase protein RhoA. RhoA reorganizes the actin cytoskeleton in myelinating SCs through interactions with actin-binding proteins Cofilin1 and myosin-II, contributing to SC reprogramming (Wen et al., 2018; Liu et al., 2023). Moreover, RhoA indirectly activates the JNK/c-Jun pathway, regulating the transcription of myelin-related genes in SCs during the reprogramming. The JNK/c-Jun pathway is typically activated by the growth factor NGF, which binds to Trk and p75NTR receptors, as shown in Figure 1 (Majdan et al., 2001; Sofroniew et al., 2001; Agthong et al., 2006; Li et al., 2020). NGF also regulates SCs’ reprogramming by rapidly activating the RAS/ERK pathway (Cervellini et al., 2018; Wen et al., 2022), which plays a role in both myelinating and non-myelinating SC phenotypes (Castelnovo et al., 2017). According to Cervellini et al., the dual functionality of this pathway relies on transient activation of phosphorylated ERK to regulate the myelination properties of SCs and enhance successful regeneration (Agthong et al., 2006; Cervellini et al., 2018).

The crosstalk between axons and SCs is also regulated by cholinergic receptors, these receptors respond to neurotransmitters such as Acetylcholine (ACh), Glutamate, γ-aminobutyric acid (GABA), and Adenosine/ATP. In particular, the M2 muscarinic receptors and the α7 nicotinic acetylcholine receptors (α7 nAChRs) influence SC plasticity and function in PNR (Piovesana et al., 2022). Activation of these α7 nAChRs is facilitated by neurotransmitter ACh, which is expressed and released by motor and sensory nerves, further suggesting its involvement in the SC-axon crosstalk. Intriago et al. reported an increase in α7 nAChRs expression in SCs 24 h following PNI, which reduced the expression of proinflammatory IL-6 cytokine, hence, regulating the inflammatory response. Furthermore, the increase in α7 nAChRs expression correlates with a decrease in c-Jun expression, which is a transcription factor known to mediate SC plasticity (Salazar Intriago et al., 2021; Piovesana et al., 2022).

**Figure 2:**
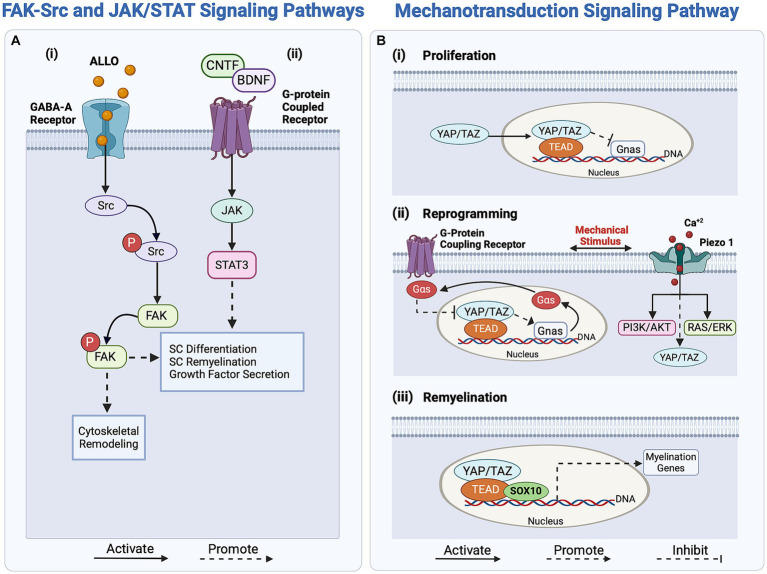
Overview of regulatory signaling pathways activating Schwann cells (SCs) response in peripheral nerve regeneration (PNR). **(Ai)** The FAK-Src pathway regulates SC proliferation and reprogramming through the binding of ALLO to the GABA-A receptor. **(ii)** The JAK/STAT signaling pathway enhances axonal outgrowth in PNR by upregulating growth factor secretion. Activated by growth factors CNTF and BDNF, this pathway enhances SC redifferentiation through the transcription of differentiation-driving genes. **(B)** The mechanotransducive signaling pathway regulates SC proliferation, reprogramming, and myelin expression. **(i)** SC proliferation is regulated by the inhibition of the protein Gnas. **(ii)** Mechanical stimuli activate G-protein coupling receptors and Piezo1 in SCs, regulating YAP/TAZ and promoting SC reprogramming by regulating the transcription of myelin genes. **(iii)** For SC redifferentiation, YAP/TAZ, along with effectors TEAD and SOX10, promote the transcription of myelin genes, driving the switch to the pro-myelinating phenotype. ALLO, Allopregnanolone; NT3, neurotrophin-3; BDNF, brain-derived neurotrophic factor; JAK, Janus Kinase; Src, proto-oncogene tyrosine-protein kinase; YAP, Yes-associated protein; TAZ, transcriptional co-activator with a PDZ-binding motif; TEAD, TEA domain family member; Gnas, guanine nucleotide-binding protein; Gαs, Gs alpha guanine nucleotide-binding signal transduction protein; SOX10, SRY-box transcription factor 10.

The reprogramming of SCs is also regulated through the activation of focal adhesion kinase (FAK) and proto-oncogene tyrosine-protein kinase (Src; Grove et al., 2007; Melfi et al., 2017). FAK, a non-receptor tyrosine kinase, promotes cell motility and actomyosin contractility to prevent premature SC differentiation and impaired nerve myelination (Grove et al., 2007; Grove and Brophy, 2014; Melfi et al., 2017). In contrast, Src is a non-receptor kinase involved in regulating various regulatory signaling pathways, including the JNK, ERK, and PI3K/Akt/mTOR pathways (Melfi et al., 2017). Zhao et al. reported that the significantly increased Src levels in SCs after injury promote axonal elongation through a crosstalk between regenerating axons and SCs (Zhao et al., 2003; Melfi et al., 2017). The activation of FAK/Src in SCs involves GABA-A-dependent mechanisms, as the binding of the neuroactive steroid Allopregnanolone (ALLO) to the GABA-A receptors expressed by SCs leads to a series of downstream intracellular signaling pathway activation, one of which is FAK/Src, as illustrated in Figure 2Ai (Colciago et al., 2020; Piovesana et al., 2022). ALLO is additionally found to upregulate SC proliferation *in vitro* following the binding of GABA-A and the modulation of excitatory amino acid transporter 1 (EAAC1). The modulatory role of ALLO is demonstrated through the GABA-A-mediated activation of protein kinase C (PK-C), leading to rapid upregulation of EAAC1 and Src levels in SCs, followed by increased EAAC1 exocytosis and modification of SC morphology (Perego et al., 2012; Piovesana et al., 2022). This increase in Src levels results in an increase of p-FAK signaling, which drives SC proliferation. As shown in Figure 2Ai, the GABA-A dependent activation mechanism of Src drives FAK phosphorylation and thereby influences actin cytoskeletal organization, migration, and proliferation of SCs (Melfi et al., 2017; Serrano-Regal et al., 2020). The FAK-Src pathway simultaneously regulates SC proliferation and reprogramming, responding to intricate biochemical signaling arising from nerve injury.

Biomechanical cues, driven by stress, strain, or changes in ECM stiffness, induce changes in the F-actin cytoskeleton of SCs, thereby influencing myelin gene transcription during their reprogramming (Fernando et al., 2016; Poitelon et al., 2016; Deng et al., 2017; Jeanette et al., 2021). External mechanical stimuli transduce into internal biochemical signals through the transcription factor Yes-associated protein (YAP) and the transcriptional co-activator with a PDZ-binding motif (TAZ), as shown in Figure 2Bi. Subsequently, YAP and TAZ regulate SC proliferation and myelin-associated gene expression via the mechanotransducive Hippo pathway (Meng et al., 2016; Deng et al., 2017; Jeanette et al., 2021). These coactivators exert distinct effects on the regenerative cascade; while TAZ levels increase following nerve injury, YAP levels remain unaffected (Mindos et al., 2017; Jeanette et al., 2021). This implies that YAP alone does not significantly affect regeneration and is not a crucial factor in SC reprogramming (Grove et al., 2020; Jeanette et al., 2021). In contrast, TAZ initiates SC reprogramming by downregulating EGR2, which inversely upregulates c-Jun expression, ensuring myelin breakdown (Parkinson et al., 2008; Jeanette et al., 2021). Interestingly, the YAP/TAZ pathway similarly regulates the remyelination phenotype of SCs (Mindos et al., 2017; Grove et al., 2020; Jeanette et al., 2021).

Other tangential regulators of the mechanotransducive signaling pathways are the mechanosensitive ion channels Piezo1 and Piezo2 which are expressed in SCs and are activated upon membrane stretching and deformation, as reported by Acheta et al. (2022). In response to injury, the mechanically gated Piezo1-2 ion channels respond to calcium bursts, regulating transient calcium levels in SCs and subsequently influencing myelin gene expression (Pathak et al., 2014; Cobbaut et al., 2020; Acheta et al., 2022). The activation of these Piezo1-2 channels may be associated with SC reprogramming, as they also mediate the YAP/TAZ regulatory pathway. Notably, Piezo1 modulates TAZ activity and expression, thereby influencing SC myelination (Acheta et al., 2022). Piezo1 is believed to inhibit myelination, whereas Piezo2 contributes to myelination, indicating a potentially epistatic relationship (Taberner et al., 2019; Acheta et al., 2022). However, limited knowledge exists about the molecular mechanisms involving Piezo2 in YAP/TAZ activation during regeneration. In contrast, inhibiting Piezo 1 has been shown to increase the activity of both ERK and AKT, which upregulate the PI3K/AKT and RAS/ERK pathways and effectively promote SC myelination, as illustrated in Figure 2Bii (Acheta et al., 2022). Thus, the Piezo1-2 channels are not only associated with the initial stages of regeneration and SC reprogramming but also play a role in shaping the remyelinating phenotype of SCs during axonal elongation and remyelination.

### 2.2 Myelin clearance

Reprogrammed SCs facilitate myelin clearance through autophagy of myelin segments (Gomez-Sanchez et al., 2015; Jessen and Mirsky, 2016). The myelin sheath structure consists of adjoining myelin segments connected to SCs through the Schmidt-Lanterman incisures. These incisures are hydrolyzed and broken down by a set of enzymes, mainly phospholipase-A2 (PLA2), which is rapidly expressed in nerve cells in response to peripheral nerve injury (Jung et al., 2011). PLA2 activates actin polymerization, leading to the separation of myelin segments from reprogrammed SCs for clearance (Jung et al., 2011; Jessen and Mirsky, 2016; Tricaud and Park, 2017). These reprogrammed SCs recruit both resident and hematogenous bone-marrow-derived macrophages to assist in myelin clearance by upregulating PLA2 and cytokines such as tumor necrosis factor-alpha (TNFα), interleukin-1α (IL-1α), IL-1β, IL-6, leukemia inhibitory factor (LIF), and monocyte chemotactic protein 1 (MCP-1; De et al., 2003; Chen et al., 2007; Martini et al., 2008; Rotshenker, 2011; Hur and Saijilafu, 2012; Mokarram et al., 2012; Defrancesco-Lisowitz et al., 2015).

Following axonal debris clearance, M1 macrophages upregulate the expression of the multifunctional surface protein galectin-3/MAC-2. Collaborating with apolipoprotein-E, galectin-3/MAC-2 mediates the polarization of macrophages into the M2 phenotype, which is considered predominant in myelin phagocytosis (Martini et al., 2008). This phenotypic transformation is associated with the downregulation of inflammatory agents TNFα and IL-1β, as well as the upregulation of anti-inflammatory cytokines IL-10 and IL-6 (Rotshenker, 2011). As degraded myelin is substantially cleared, galectin-3/MAC-2 expression is downregulated. Simultaneously, the production of anti-inflammatory cytokines and PLA2 decreases, signaling the transition from Wallerian degeneration to the axonal regeneration stage and reaching its lowest production rate approximately 2–3 weeks post-injury (De et al., 2003; Rotshenker, 2011).

### 2.3 Growth cone outgrowth and axonal elongation

Growth cone formation and axonal elongation have been associated with the PI3K/pAkt/mTOR signaling pathway (Agthong et al., 2006; Hur and Saijilafu, 2012; Menorca et al., 2013; Poitras and Zochodne, 2022). To initiate this pathway, neurotrophic molecules bind to Trk receptors, transducing signals through the cellular membrane. This pathway is controlled by the mTOR effector, which facilitates PNR. Downstream activation of mTOR complex-1 (mTORC1), a mediator of growth factor signaling, occurs via Akt-mediated phosphorylation to ensure cell survival and neuronal growth, as shown in Figure 1 (Aoki and Fujishita, 2017; Abe et al., 2021; Poitras and Zochodne, 2022). The PI3K/Akt/mTOR pathway must be tightly regulated, as its overactivation can lead to uncontrolled cellular proliferation and tumor formation instead of regulated regeneration (Liu et al., 2014; Aoki and Fujishita, 2017; Poitras and Zochodne, 2022).

The RhoA/ROCK pathway acts as a negative regulator of growth cone outgrowth and axonal elongation, as its activation leads to growth cone collapse. During axonal elongation, growth factors, including nerve growth factor (NGF) and glial cell line-derived neurotrophic factor (GDNF), are recruited to inhibit the RhoA/ROCK pathway, as illustrated in Figure 1 (Yoong and Too, 2007; Li et al., 2020). Additionally, blocking ROCK activity both *in vivo* and *in vitro* has shown enhanced axonal sprouting, which presents a plausible clinical therapeutic application (Huelsenbeck et al., 2012; Rohrbeck et al., 2015; Li et al., 2020). For instance, the local application of *Clostridium botulinum* C3 exoenzyme (C3), a RhoA inhibitor, in a rat model resulted in increased axonal sprouting, higher myelination, and enhanced axonal maturation and functionality, further hinting at the negative regulation of the RhoA/Rock pathway in PNR (Huelsenbeck et al., 2012; Penna et al., 2012; Rohrbeck et al., 2015). ROCK inhibitors are considered promising treatment options for corneal wound healing and endothelial regeneration (Okumura et al., 2011, 2012). Given their ability to expedite and enhance axonal sprouting, the applications of ROCK inhibitors can be extended to PNR.

To promote axonal growth, intracellular molecular mechanisms, such as regeneration-associated gene expression and growth cone protein synthesis, are upregulated. Post-transcriptional mechanisms related to regeneration-associated gene promote and accelerate axonal elongation through the activation of the RAS/ERK pathway (Avruch, 2007; Hausott and Klimaschewski, 2019). As shown in Figure 1, the RAS/ERK pathway activates the transcription of neuronal genes and the synthesis of growth-associated proteins, mainly growth-associated protein-43 (GAP43), cytoskeleton-associated protein-23 (Cap23), arginase-1 (Arg1), and small proline-rich repeat protein-1A (Sprr1a). These growth-associated proteins initiate the polymerization of actin filaments and microtubules, driving axonal outgrowth extension (Frey et al., 2000; Bomze et al., 2001; Bonilla et al., 2002; Cai et al., 2002). The structure of a growth cone is highly dependent on actin filaments and microtubules for its integrity. Inhibition of ERK has been associated with growth cone collapse, resulting in axonal outgrowth impairment (Bonilla et al., 2002; Goold and Gordon-Weeks, 2005; Zhou and Snider, 2006). Moreover, in a non-regenerative state, sprouty homolog-2 (SPRY-2), a protein expressed in the PNS, binds to growth factor receptor-bound protein 2 (GRB2) and inhibits ERK activation. Following peripheral nerve injury, SPRY-2 expression is downregulated and promotes axonal regeneration both *in vivo* and *in vitro*, highlighting the regulatory role of the RAS/ERK pathway. The activation of ERK and the downregulation of SPRY-2 are associated with improved growth cone structure and axonal outgrowth and can contribute to neuronal survival and axonal recovery (Marvaldi et al., 2015; Huang et al., 2017; Hausott and Klimaschewski, 2019).

Concurrently, both non-myelinating and myelinating SCs differentiate into repair phenotypes to promote axonal elongation. This differentiation process involves the upregulation of c-Jun expression, illustrated in Figure 1, leading to repair SCs adopting a narrow and flattened morphology, essential for the formation of the bands of the Büngner, a longitudinal guidance track (Arthur-Farraj et al., 2012; Jessen and Mirsky, 2016). Arthur-Farraj et al. reported abnormal bands of Büngner structures in c-Jun mutant mouse models, further emphasizing the substantial role of c-Jun in PNR (Arthur-Farraj et al., 2012; Jessen and Mirsky, 2016, 2021). This guidance track, with the help of fibroblasts and the ECM structure, ensures cohesive axonal elongation and the successful integration of regenerated nerves with the target organ tissue.

While the role of fibroblasts in axonal elongation is vital, it has received little attention in the past. He et al. (2022) recently examined the genetic expression in nerve fibroblasts, revealing their influence on axonal outgrowth and directional guidance. These fibroblasts express brain-derived neurotrophic factor (BDNF), which upregulates the expression of β-actin and F-actin through the RAS/ERK and PI3K/pAkt pathways, thereby promoting axonal outgrowth and neural survival, as shown in Figure 1 (Garraway and Huie, 2016; Weiss et al., 2016; Moradi et al., 2017; He et al., 2022). In addition to supporting axonal outgrowth, fibroblasts play a vital role in recruiting repair SCs, contributing to the formation of the supportive Bands of Büngner. The migration of recruited SCs is regulated by several factors, mainly neuregulin-1b1 and tenascin-C (TNC; Dreesmann et al., 2009; van Neerven et al., 2013; He et al., 2022; Li et al., 2022). TNC induces SC migration through the β-1 integrin-dependent signaling pathway (Dreesmann et al., 2009; Zhang et al., 2016; He et al., 2022; Li et al., 2022). The binding of TNC to the β-1 integrin on the surface of SCs activates Rac1, a regulator of the RAS/ERK pathway, which, in turn, promotes SC migration and enhances the structural stability of the regenerative microenvironment (Zhang et al., 2016).

Another pathway contributing to neuronal plasticity, axonal guidance, and regeneration is the JAK/STAT, as shown in Figure 2Aii. Unlike many regulatory pathways, JAK/STAT is not activated by neurotrophic Trk receptors, but rather by a G-protein-coupled receptor that binds neurotrophic factors, such as ciliary neurotrophic factor (CNTF), NT3, and BDNF, to initiate the signaling cascade (Sheu et al., 2000; Qiu et al., 2005; Kiryu-Seo and Kiyama, 2011; Poitras and Zochodne, 2022). These neurotrophic factors upregulate STAT3 in SCs, which subsequently enhances growth factor secretion and neurite sprouting (Aoki and Fujishita, 2017; Poitras and Zochodne, 2022).

In addition, the local release of ACh at the site of injury serves as a direct trigger for growth cone outgrowth. For instance, when introducing ACh-loaded biodegradable polymers to facilitate the release of ACh at the site of injury, increased nerve sprouting and elongation was observed, further hinting at the role of the cholinergic system in aiding PNR (Gumera and Wang, 2007; Magnaghi et al., 2009). Thus, in addition to the PI3K/pAkt/mTOR, RhoA/ROCK, RAS/ERK, and JAK/STAT signaling pathways, the cholinergic system orchestrates nerve outgrowth and elongation.

### 2.4 Newly generated nerve myelination and integration

To conclude the regenerative cascade, both repair and non-myelinating SCs undergo redifferentiation to myelinate the newly regenerated nerve. This redifferentiation process is associated with the upregulation of myelin transcription factors, specifically neuregulin 1 (NRG1), and the downregulation of repair-related genes such as oligodendrocyte transcription factor 1 (Olig1), Sonic Hedgehog (Shh), GDNF, and c-Jun (Piirsoo et al., 2010; Arthur-Farraj et al., 2012; Benito et al., 2017; Bosch-Queralt et al., 2023). As mentioned earlier, the YAP/TAZ regulatory mechanism contributes to this redifferentiation (Figure 2Biii). YAP/TAZ activation upregulates EGR2 levels, leading to a decrease in c-Jun levels and an increase in myelinating gene expression (Jeanette et al., 2021). Upon activation, YAP and TAZ form multiple complexes with DNA-binding proteins, including TEAD1, which is associated with SC redifferentiation, and TEAD4, which represses SC myelination. During redifferentiation, the expression of myelin-related genes is regulated by TEAD1, driving SC remyelination (Lopez-Anido et al., 2016; Deng et al., 2017; Grove et al., 2017; He et al., 2018; Jeanette et al., 2021).

Simultaneously, as shown in Figure 2Aii, the JAK/STAT pathway regulates SC redifferentiation and migration alongside the elongated axon. To activate this pathway, neurotrophic factors such as CNTF, IL-6, and LIF bind to their receptors, which depends on their specific subunit composition, leading to STAT3 activation (Martini et al., 2008). Consequently, the JAK/STAT pathway activates the transcription of differentiation-driving genes (stemness-related genes) to initiate the redifferentiation of SCs (Birchmeier and Bennett, 2016; Jessen and Arthur-Farraj, 2019; Li et al., 2020; Jessen and Mirsky, 2021). Alongside the JAK/STAT pathway, the NRG1-ErbB signaling pathway promotes SC proliferation, myelination, and migration along the axon tract. More specifically, type I NRG1 and type III NRG1, found in SCs and nerve cells respectively, activate the ErbB2/3 protein-ligand interaction, promoting SCs differentiation and remyelination (Atanasoski et al., 2006; Fricker and Bennett, 2011; Fricker et al., 2013). Following the disruption of the SC-axon contact, NRG1 type I expression in SCs is upregulated, activating the NRG1-ErbB signaling pathway and instigating the remyelination of axons by regulating myelin gene transcription (Fricker and Bennett, 2011; Stassart et al., 2013). In addition, the activation of the NRG1 through the ErbB2/3 receptors propagates to promote FAK phosphorylation, leading to FAK/Src activation and subsequently, SC migration along the elongated axon (Chang et al., 2013). In essence, the myelination process is vital to ensure the successful integration of a regenerated nerve with muscle fibers at neuromuscular junctions for effective signal transduction (Aguayo et al., 1973; Tam and Gordon, 2009; Menorca et al., 2013).

In summary, the regenerative cascade involves a complex interplay of various regulatory pathways that facilitate interactions between nerve cells and other contributing cells. These pathways collectively support the survival, growth, protection, guidance, and regeneration of nerve cells. Specifically, the JNK/c-Jun, RhoA/ROCK, and JAK/STAT are key intracellular regulatory pathways involved in regeneration. Additionally, cellular communication plays a crucial role in directing the regenerative cascade, primarily by activating repair-associated gene transcription via the FAK-Src, PI3K/pAkt/mTOR, RAS/ERK, and YAP/TAZ pathways (Poitelon et al., 2016). The functions of these intracellular pathways are summarized in Table 1, highlighting their regulatory roles and the associated proteins.

**Table 1:** Summary of the regulatory signaling pathways within the regenerative cascade.

Signaling pathways	Involved cells	Associated regulators	Regulatory role	References
JNK/c-Jun	Schwann Cells	RhoA, NGF, c-Jun	Promote cell reprogramming	Melfi et al. (2017) and Wen et al. (2018)
RhoA/ROCK	Nerve Cells	RhoA, BDNF, GDNF	Inhibits axonal elongation	Yoong and Too (2007), Huelsenbeck et al. (2012), Penna et al. (2012), and Rohrbeck et al. (2015)
RAS/ERK	Nerve and Schwann Cells	NGF, BDNF	Regulates myelination and promotes growth cone outgrowth	Obata et al. (2003), Agthong et al. (2006), Marvaldi et al. (2015); Huang et al. (2017), and Cervellini et al. (2018)
JAK/STAT	Nerve and Schwann Cells	CNTF, IL-6, LIF	Promotes axonal elongation and SC remyelination	Sheu et al. (2000), Qiu et al. (2005), and Birchmeier and Bennett (2016)
NRG1-ErbB	Nerve and Schwann Cells	Type I NRG1, Type III NRG1	Promotes SC proliferation, myelination, and migration along the axon tract	Atanasoski et al. (2006), Fricker and Bennett (2011), Chang et al. (2013), and Stassart et al. (2013)
FAK-Src	Schwann Cells	Neuroactive Steroid ALLO	Promotes SC reprogramming	Grove et al. (2007), Grove and Brophy (2014), and Melfi et al. (2017)
PI3K/pAkt/mTOR	Nerve and Schwann Cells	NGF, BDNF, NT3	Promotes growth cone outgrowth Promotes SC myelination.	Agthong et al. (2006), Huang et al. (2017), and Acheta et al. (2022)
YAP/TAZ	Schwann Cells	TEAD, Piezo1, Piezo2	Promotes cell reprogramming	Grove et al. (2020), Jeanette et al. (2021), and Acheta et al. (2022)

JNK, c-Jun N-terminal kinase; c-Jun, Jun proto-oncogene; RhoA, Ras homolog family member A; ROCK, Rho-associated protein kinase; RAS, rat sarcoma; ERK, extracellular signal-regulated kinase; JAK, Janus Kinase; STAT, signal transducer and activator of transcription; FAK, focal adhesion kinase; Src, proto-oncogene tyrosine-protein kinase; PI3K, phosphoinositide 3-kinase; AKT, Protein kinase B; mTOR, mammalian target of rapamycin; YAP, Yes-associated protein; TAZ, transcriptional co-activator with a PDZ-binding motif; BDNF, brain-derived neurotrophic factor; NGF, nerve growth factor; GDNF, glial cell line-derived neurotrophic factor; CTNF, ciliary neurotrophic factor; IL-6, interleukin-6; LIF, leukemia inhibitory factor; NRG1, Neuregulin 1; ALLO, Allopregnanolone; NT3, neurotrophin-3; TEAD, TEA domain family member; SC, Schwann cell.

## 3 Extracellular vesicles and their role in peripheral nerve regeneration

In addition to signaling pathways, it is important to explore intercellular communication via EVs, specifically sEVs, and their role in regulating communication within the regenerative cascade. EVs are membrane-bound vesicles containing proteins, lipids, nucleic acids, and metabolites that mediate intercellular communication. EVs are classified based on several factors, such as biogenesis, size, and cargos, which help identify their originating source and type (Zernecke et al., 2009; Kosaka et al., 2010; Vickers et al., 2011; Jin et al., 2016; Kodam and Ullah, 2021). These classifications include exomeres (<50 nm diameter), exosomes (30–150 nm diameter), ectosomes or microvesicles (MVs; 100–1,000 nm diameter), migrasomes (500–3,000 nm diameter), apoptotic bodies (1000–5,000 nm diameter), and large oncosomes (1000–10,000 nm diameter; Fonseka et al., 2021). Exosomes are sEVs formed by the inward budding of endosomal membranes to form intraluminal vesicles (ILVs) encapsulating cytosolic components (Kodam and Ullah, 2021). In contrast, ectosomes, apoptotic bodies, and large oncosomes are medium to large-sized EVs formed through direct budding from cellular membranes (Akers et al., 2013; Raposo and Stoorvogel, 2013; Hercher et al., 2022).

### 3.1 Biogenesis of extracellular vesicles

sEV biogenesis involves ILVs coming together to form multivesicular bodies (MVBs), which can either be degraded by lysosomes or released from the cell as sEVs through the endosomal sorting complex required for transport (ESCRT) regulatory system (Qing et al., 2018; Fonseka et al., 2021; Gurung et al., 2021). The secretion of sEVs is regulated by four ESCRT protein complexes: ESCRT-0, I, II, and III. The ESCRT-0 complex aids in identifying and trafficking cargo within the endosomal membrane. In contrast, ESCRT-I and -II control cargo sorting and vesicle bud formation, while ESCRT-III functions as a membrane scission machine to cleave buds and form ILVs (Wollert et al., 2009; Hurley and Hanson, 2010; Colombo et al., 2013; Hessvik and Llorente, 2018).

ESCRT protein complexes are associated with regulatory proteins that contribute to ILV sorting, such as hepatocyte growth factor-regulated tyrosine kinase substrate (HRS), tumor susceptibility gene 101 (TSG101), and ALG-2 interacting protein X (ALIX; Colombo et al., 2013). ALIX has been recently identified to be directly involved in ILV budding, sEV biogenesis, and cargo sorting (Baietti et al., 2012; Colombo et al., 2013; Gurung et al., 2021). Moreover, sEVs contain tetraspanins, a family of proteins with transmembrane domains associated with cellular functions, such as adhesion and cellular signaling. Tetraspanins are also involved in the ESCRT-independent mechanism of sEV biogenesis (Termini and Gillette, 2017; Gurung et al., 2021; Bischoff et al., 2022). Some tetraspanins, such as CD9, CD63, and CD81 are used as specific markers to identify sEVs (Chairoungdua et al., 2010; Urbanelli et al., 2013). Additionally, sEVs encapsulate noncoding RNAs, such as miRNAs, which play roles in regulating gene expression, intracellular signaling, and protein expression (Lewis et al., 2005; Ching and Kingham, 2015; Budnik et al., 2016; van Niel et al., 2018; Borger et al., 2022).

### 3.2 Role of small extracellular vesicles in intercellular communication

Cell-to-cell communication via sEVs may occur through three distinct mechanisms, one of which is receptor-ligand signaling, where membrane-bound proteins of sEVs act as ligands for surface receptors on recipient cells. Another communication mechanism involves the release of the sEV cargo into the extracellular space (Gurung et al., 2021), facilitating indirect communication between cells, as the released proteins bind to recipient cell surface receptors to induce an internal regulatory response. Alternatively, sEVs can be internalized by recipient cells, resulting in nonselective cargo release, which activates internal signaling pathways, including gene transcription (Urbanelli et al., 2013).

Besides facilitating intercellular communication, sEVs encapsulate proteins involved in internal regulatory pathways, such as the Wnt/β-catenin pathway, known for its role in tissue regeneration (Mac Donald et al., 2009; Urbanelli et al., 2013; Budnik et al., 2016). Wnt proteins embedded within sEV membranes activate the Wnt signaling pathway by binding to the seven-pass transmembrane fizzled receptors (FZ) on the surface of target cells (Mac Donald et al., 2009; Urbanelli et al., 2013). In principle, this regulatory process activates Wnt gene expression and regulates sEV-mediated transport of Wnt via a feedback mechanism (Mac Donald et al., 2009; Chairoungdua et al., 2010; Urbanelli et al., 2013). While these findings elucidate the relationship between sEVs and Wnt signaling, the extent to which the peripheral nerve regenerative cascade activates Wnt signaling via sEV communication remains underexplored.

### 3.3 Small extracellular vesicles in peripheral nerve regeneration

**Figure 3:**
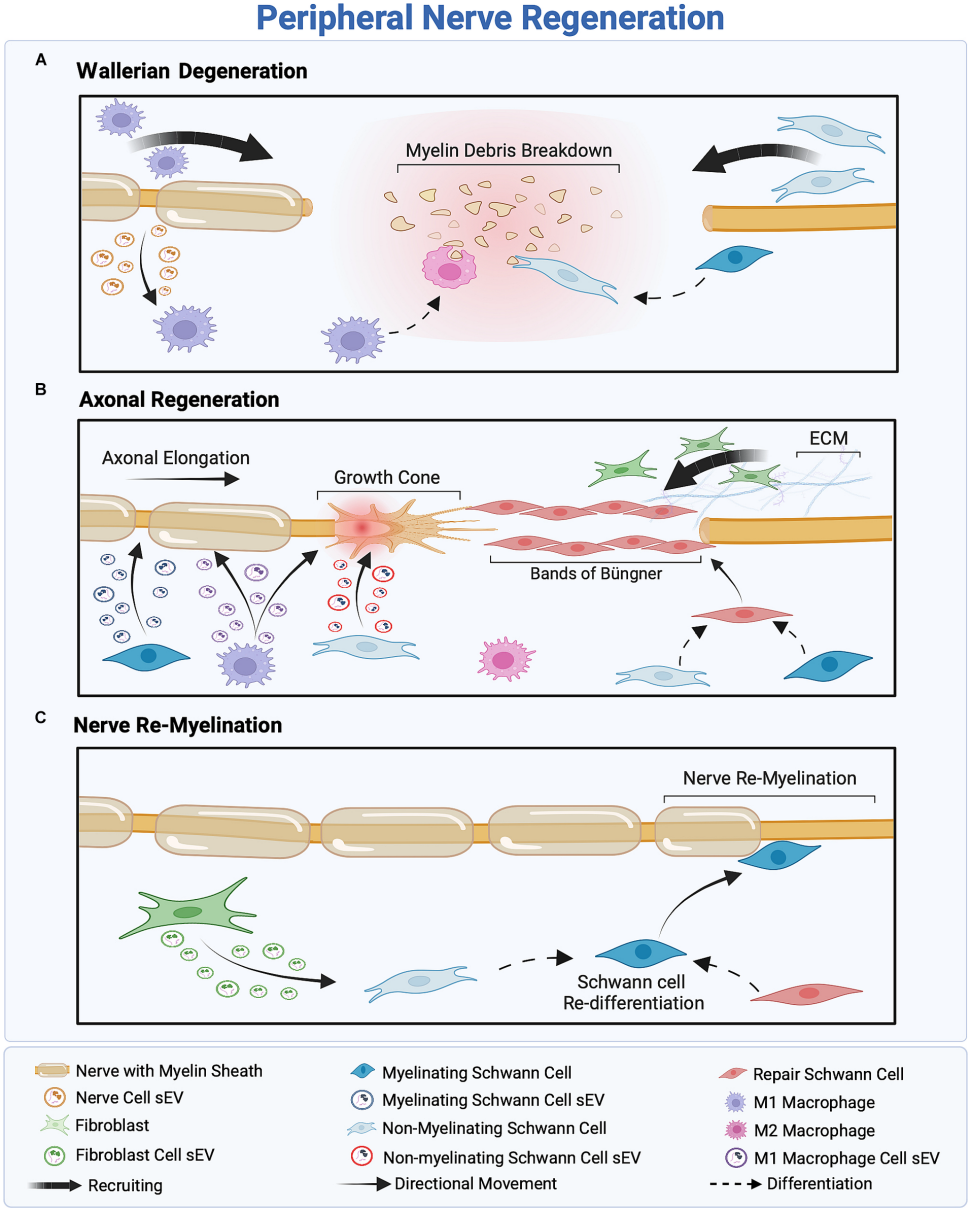
Overview of the three-stage peripheral nerve regeneration (PNR). **(A)** Wallerian degeneration orchestrates the clearance of cellular and myelin debris, facilitated by the recruitment of macrophages and Schwann cells (SCs). SCs inhibit myelin production and reprogram into non-myelinating SCs to eliminate remnants of the injured nerve, while M1 macrophages scavenge cellular debris and regulate inflammation. **(B)** The proximal part of the injured nerve forms a growth cone, inducing axonal outgrowth. Fibroblast recruitment provides structural support to regenerated axons, facilitating SC differentiation into the repair phenotype and the formation of Bands of Büngner as a guidance track. Macrophage-and SC-derived small extracellular vesicles (sEVs) promote axonal outgrowth through intercellular communication within the regeneration cascade. **(C)** Assisted by fibroblast-derived sEVs, SCs redifferentiate into the myelinating phenotype to remyelinate the nerve, ensuring successful regeneration.

sEVs derived from most PNS-associated cells have been reported to exert regenerative effects (Dong et al., 2019; Bischoff et al., 2022; Hercher et al., 2022). However, the intricate communication mechanisms influencing sEV secretion within the regenerative cascade remain poorly understood. Cellular stress conditions, such as oxidative stress, enhance sEV secretion (Faure et al., 2006; Yu et al., 2006; Urbanelli et al., 2013; Qiu et al., 2019). Following peripheral nerve injury, the stress-responsive protein p53 regulates sEV secretion through the transcription of sEV secretion-related genes such as TSAP6 (Zhou et al., 2005; Yu et al., 2006; Pegtel et al., 2014). According to Simeoli et al., nerve cell-derived sEVs are phagocytosed by macrophages after nerve injury, as illustrated in Figure 3A, initiating miR-21-5p upregulation (Simeoli et al., 2017; Qing et al., 2018; Liu et al., 2019; Bischoff et al., 2022). Upregulation of miR-21-5p induces the uptake of inducible nitric oxide synthase (iNOS), a pro-inflammatory M1 marker, also known as Nos2, thereby promoting the activation of pro-inflammatory M1 macrophages. Simultaneously, to promote the M1 phenotype, sEVs secreted by injured nerves downregulate CD206 mRNA expression, leading to a decrease in the expression of anti-inflammatory M2 markers (Simeoli et al., 2017). This sEV-mediated nerve–macrophage communication serves as a regulator of macrophage response and axonal outgrowth. Along the regenerative cascade, sEVs derived from pro-inflammatory macrophages mediate the PI3K/Akt signaling pathway, as they promote nerve outgrowth by encapsulating active NADPH oxidase 2 (NOX2), as illustrated in Figure 3B (Hervera et al., 2018; Bischoff et al., 2022).

Conversely, sEVs secreted by SCs play a crucial role in intercellular communication between nerve cells and SCs following nerve injury. Wei et al. (2019) reported that SC-derived sEVs, regardless of the SC phenotype, typically express sEV markers CD9, CD63, and ALIX, but not TSG10 (). These markers facilitate the accurate identification of sEVs’ cellular origins and an understanding of their role in intercellular communication within the regenerative cascade. Notably, different SCs phenotypes—myelinating, non-myelinating, and repair SCs—release sEVs with distinct cargo profiles, illustrated in Figure 3B (Lopez-Verrilli et al., 2013; Sohn et al., 2020; Bischoff et al., 2022). For instance, sEVs derived from non-myelinating SCs encapsulate the protein P75NTR, which inhibits RhoA and promotes growth cone outgrowth (Budnik et al., 2016; Lopez-Leal and Court, 2016). In contrast, sEVs derived from myelinating SCs express high levels of miR92a-3p, which modulates the Akt signaling pathway and upregulates neurite outgrowth (Sohn et al., 2020). Additionally, sEVs secreted from repair SCs encapsulate high levels of miRNA-21, which promotes axonal outgrowth (Ching and Kingham, 2015; Lopez-Leal et al., 2020). Regardless of their source—myelinating, non-myelinating, or repair SCs—sEVs have a significant impact on axonal elongation, attributed to distinct proteins and miRNAs associated with each phenotype.

Previously, fibroblast-derived sEVs were believed to have no effect on the peripheral nerve regenerative cascade; however, recent findings indicate the presence of an sEV-mediated crosstalk between fibroblasts and SCs, as illustrated in Figure 3C (Bischoff et al., 2022). Zhao et al. (2022) reported that fibroblast-derived sEVs carry miR-673-5p, which targets the *Tsc2* gene, which encodes tuberin, a protein contributing to cellular growth and proliferation. This gene has been found to activate the mTORC1 growth factor mediator in SCs, thereby activating the PI3K/Akt/mTOR pathway to promote myelin gene expression (Zhao et al., 2022). Moreover, fibroblast-derived sEVs stimulate cholesterol and lipid synthesis in SCs, both of which promote myelin formation, as illustrated in Figure 3C (Zhao et al., 2022). These fibroblast-derived sEVs can be characterized by the sEV markers ALIX, CD9, CD81, and Flotillin-1 and, more specifically, by the surface proteins CD63 and CD44 (Mead and Tomarev, 2017; van de Vlekkert et al., 2019, 2020; Yan et al., 2020). Table 2 summarizes the roles of sEVs in regulating intercellular communication during PNR.

**Table 2:** Summary of the small extracellular vesicle communication involved in the regenerative response.

Target cell	Cell of origin	Signaling pathways involved	Regenerative role	References
Nerve Cell	Myelinating Schwann Cell	PI3K/Akt signaling pathway	Enhances neurite outgrowth	Sohn et al. (2020)
	Non-myelinating Schwann Cell	RhoA GTPase inhibition	Regulates growth cone formation	Lopez-Verrilli et al. (2013)
	Repair Schwann Cell	Upregulation of miRNA-21 transcription	Enhances neurite outgrowth	Lopez-Leal et al. (2020)
	M1 Macrophage Cell	PI3K/Akt signaling pathway	Promote nerve outgrowth	Hervera et al. (2018)
M1 Macrophage Cell	Nerve Cell	Upregulation of miR-21-5p transcription	Promotes a proinflammatory phenotype	Simeoli et al. (2017)
Schwann Cell	Fibroblast Cell	Activation of mTORC1	Promotes nerve remyelination	Zhao et al. (2022)

PI3K, phosphoinositide 3-kinase; AKT, Protein kinase B; RhoA, Ras homolog family member A; mTORC1, mammalian target of rapamycin complex 1.

Intercellular communication through sEVs has garnered significant attention owing to its wide-ranging therapeutic and diagnostic potential in the context of PNR. Ongoing research on the therapeutic potential of sEVs in PNI extends beyond studies focused solely on sEVs derived from PNS-associated cells. For instance, sEVs secreted from diverse sources of mesenchymal stem cells (MSCs), such as bone marrow, umbilical cord, adipose tissue, and dental pulp, have shown the capacity to enhance neural growth (Qing et al., 2018). Notably, the uptake of MSC-derived sEVs by SCs, both *in vivo* and *in vitro*, has been reported to enhance SC migration and proliferation, macrophage activation, and axonal outgrowth (Triolo et al., 2006; Ti et al., 2015; Bucan et al., 2019; Dong et al., 2019; Mao et al., 2019; Bischoff et al., 2022). Collectively, MSC-derived sEVs have been found to activate the PI3K/Akt regulatory pathway, as well as the ERK and STAT3 signaling pathways, thereby enhancing the secretion of growth factors that contribute to axonal regeneration and outgrowth (Shabbir et al., 2015; Qing et al., 2018; Dong et al., 2019).

Similar to sEVs, MVs have shown a promising role in enhancing PNR by facilitating the transport of cytoskeletal proteins and RNAs between cells. Treatment of peripheral nerve injury *in vivo* with MSC-derived MVs has been found to enhance axonal growth by regulating the PTEN/PI3K/Akt signaling pathway (Ye et al., 2021). Additionally, M2 macrophage-derived MVs upregulate the expression of miR-233, which increases laminin expression and NGF secretion, resulting in enhanced nerve regeneration (Zhan et al., 2015). In contrast, the role of M1-derived MVs in PNR has not been extensively explored; however, they have been investigated as a therapeutic strategy for cardiovascular diseases and an inhibitory factor for tumor progression in colon cancer (Huis In Veld et al., 2022; Xia et al., 2022). Despite these findings, there remains a lack of understanding regarding the molecular mechanisms of intercellular communication through MVs, which creates a gap in our ability to fully assess the efficacy of MVs in promoting PNR (Zhan et al., 2015; Hercher et al., 2022).

The transportation of miRNAs through sEVs and MVs is pivotal for regulating cellular communication within nerve cells, SCs, macrophages, fibroblasts, and MSCs. Table 3 provides an overview of the roles of miRNAs in regulating biological functions, including axonal growth, cell migration, and cell proliferation, within the PNS.

**Table 3:** Role of miRNAs in regulating the regenerative response.

Cell of origin	Micro-RNA	Biological function	Signaling pathway	EVs involved	References
Schwann Cell	miR-340	Regulates debris removal and axonal outgrowth	Inhibiting TPA protein expression	Unreported	Li et al. (2017)
	miR-221/222	Promotes Schwann Cell migration and proliferation	PI3K/Akt/mTOR	sEVs	Yu et al. (2012)
	miR363	Suppresses Schwann Cell migration	RAS/ERK signaling pathway	sEVs	Sohn et al. (2020)
	miR22-3p				
	miR29a-3p		Wnt signaling pathway		
	miR-21	Accelerates axonal outgrowth	Akt signaling pathway	sEVs	Liu et al. (2022)
Nerve Cell	miRNA-132	Promotes nerve growth.	cAMP signaling pathway	sEVs	Qian et al. (2017)
			ERK-dependent CREB signaling cascade		
	miR-21-5p	Shift macrophage phenotype toward M1 pro-inflammatory	Unreported	sEVs	Simeoli et al. (2017)
	miRNA-21	Promotes Schwann Cell proliferation and nerve regeneration	Regulating TGFβI, TIMP3 and EPHA4 target genes	sEVs	Lopez-Leal et al. (2020) and Ning et al. (2020)
	miRNA Let-7	Regulates Schwann Cell proliferation and migration	NGF-independent pathway	Unreported	Li et al. (2015)
			Inflammatory cytokines (IL-6 and IL-10)		
M2 Macrophage	miR-223	Promotes Schwann Cell migration, proliferation, and axonal outgrowth	Upregulating NGF and laminin expression	MVs	Zhan et al. (2015)
Fibroblast	miR-673-5p	Regulates Schwann Cell myelination state	Activating the mTORC1 growth factor mediator by the Tsc2 gene	sEVs	Zhao et al. (2022)
Mesenchymal Stem Cells	miRNA-17-92	Enhances axonal outgrowth	PI3K/PTEN/mTOR	sEVs	Zhang Y. et al. (2017)
	miRNA let-7b	Controls the switch in macrophage phenotype	TLR4/NF-κB/ STAT3/AKT	sEVs	Ti et al. (2015)

TPA, tissue-type plasminogen activator; PI3K, phosphoinositide 3-kinase; AKT, Protein kinase B; RhoA, Ras homolog family member A; mTOR, mammalian target of rapamycin; mTORC1, mammalian target of rapamycin complex 1; TLR4, Toll-like receptor 4; NF-κB, nuclear factor kappa-light-chain-enhancer of activated B cells; STAT3, signal transducer and activator of transcription 3; PTEN, Phosphatase and TENsin homolog deleted on chromosome 10; Tsc2, Tuberin; NGF, nerve growth factor; ERK, extracellular signal-regulated kinase; IL-6/10, interleukin-6/10; cAMP, cyclic adenosine monophosphate; CREB, cAMP response element-binding protein; TGFßI, transforming growth factor beta induced; TIMP3, TIMP Metallopeptidase Inhibitor 3; EPHA4, ephrin type-A receptor 4.

## 4 Electrical stimulation in peripheral nerve regeneration

The application of ES within the PNS has a profound influence on cell migration, proliferation, and tissue regeneration (Du et al., 2018). ES promotes regeneration within the PNS by specifically targeting nerve cells, SCs, and macrophages. Hu et al. (2019) examined the effect of direct electric current stimulation on DRG nerve cells in an electrotactic cell culture chamber. When comparing the cellular condition following electric fields of 100 and 200 mV/mm it was observed that, cell viability, proliferation, and density were higher at 100 mV/mm than at 200 mV/mm (Hu et al., 2019). Further investigation into the impact of 100 mV/mm stimulation for varying durations, ranging from half an hour to 2 h, indicated that a half-hour stimulation had no significant effect on cells, while a 2-h stimulation led to decreased viability. This reduction can be attributed to the prolonged alteration of the cell membrane depolarization and repolarization states, which ultimately affects the integrity of the membrane structure. Thus, the reported optimal parameters for ES are an intensity of 100 mV/mm ES and a duration of 1 h (Hu et al., 2019). Concerning SC stimulation, Koppes et al. found that electrically stimulating SCs at a frequency of 20 Hz (with pulses of 100 μs at 3 V) in an electrotaxis setup led to the overexpression of growth-associated protein (GAP)-43, ɑ1-tubulin, and TrkB, all of which contribute to axonal outgrowth and nerve regeneration (English et al., 2007; Geremia et al., 2007; Koppes et al., 2014; Li et al., 2023).

Various ES parameters have been reported for *in vivo* peripheral nerve injury models (Jin et al., 2023). For instance, applying ES for 1 h at 20 Hz (with pulses of 100 μs, 3–5 V) in a rat model showed rapid nerve regeneration, reducing the axonal outgrowth timeline from to 2–3 months to 3 weeks (Al-Majed et al., 2000; Brushart et al., 2005). Furthermore, McLean and Verge (2016) modeled peripheral nerve injury in rats by creating focal demyelination lesions in the sciatic nerve with an injection of 1% Lysophosphatidylcholine (LPC), which disrupts the myelin structure. Five days following injection, direct pulsed ES was performed for 1 h (with pulses of 100 ms at 3 V) and revealed expedited myelin debris clearance and enhanced regeneration by upregulating the anti-inflammatory response in macrophages (McLean and Verge, 2016). In addition, ES promotes higher specificity in directional axonal outgrowth and reduces axonal crossover, thereby improving regeneration (Brushart et al., 2002, 2005). Roh et al. (2022) used 0.5 mA stimulations at 16 Hz in a rat model to examine the interchangeable effects of treatment duration on axonal outgrowth. The group found that a short-term (14 days) application of 10-min ES increased axonal growth. However, a long-term (52 days) 10-min ES did not yield an enhanced regenerative effect (Roh et al., 2022; Jin et al., 2023). This finding raised the question of whether a shorter duration of ES is sufficient to enhance axonal growth. Calvey et al. (2015) demonstrated that ES at 1 mA and 20 Hz in rats, whether applied for 10 and 60 min, exhibited the same increase in nerve regeneration after 12 weeks of stimulation. These findings suggest a potentially safer approach to facilitate enhanced nerve regeneration and functional recovery (Calvey et al., 2015; Jin et al., 2023). As for the choice of the ideal ES frequency setting to augment regeneration, Lu et al. (2008) investigated the effect of different ES frequency settings on regeneration. When comparing the application of 1 mA percutaneous ES at 1, 2, 20, and 200 Hz frequencies in a rat model for 15 min, stimulation at a frequency of 2 Hz presented to be most successful in augmenting regeneration (Lu et al., 2008). Peripheral nerves treated with ES at 2 Hz expressed higher myelination, higher axon density, and a higher ratio of blood vessel to total nerve area (Lu et al., 2008). On the other hand, stimulation at a frequency of 200 Hz led to reduced PNR in comparison to lower frequencies, further highlighting the efficiency of low-frequency ES for regeneration. Overall, the gold standard for *in vivo* studies appears to be an ES of 20 Hz for 1 h, with voltage ranging from 0.5 to 5 V (Zuo et al., 2020; Ni et al., 2023). However, the detailed molecular mechanisms underlying the observed regenerative effects of these specific parameters remain largely unknown (Geremia et al., 2007; Gordon et al., 2007; Gordon, 2016). For comprehensive review articles that extensively explore various *in vivo* ES protocols for PNR, please refer to Pullar (2011), Gordon (2016), Willand et al. (2016), and Zuo et al. (2020).

### 4.1 Electrical stimulation to regulate intracellular signaling mechanisms

**Figure 4:**
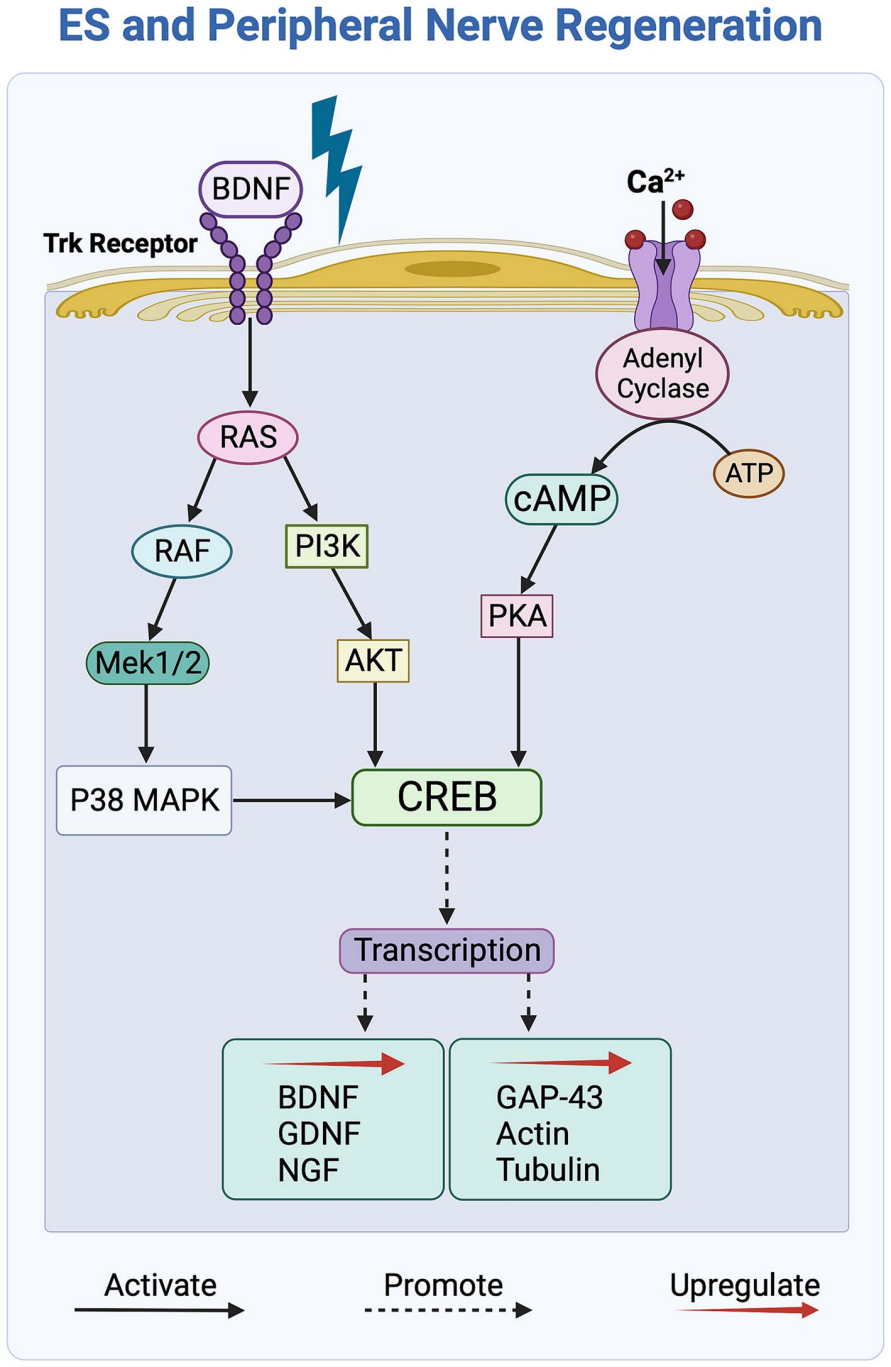
Overview of the molecular signaling pathways affected by electrical stimulation (ES) of peripheral nerves. ES activates the Trk and Ca^2+^ receptors, initiating the cAMP signaling pathway, which, in turn, upregulates the transcription of growth factors and cytoskeletal proteins to promote axonal outgrowth. The PI3K/Akt and RAS/ERK signaling pathways further contribute by activating the transcription factor CREB, which subsequently leads to an increase in the secretion of growth factors.

Exposure to ES activates the neuronal cyclic adenosine monophosphate (cAMP) pathway, a key regulatory process that contributes to enhanced axonal growth guidance and nerve outgrowth upon ES of nerve cells *in vitro*. More specifically, as shown in Figure 4, this activation is triggered by BDNF binding to the Trk receptor and an ensuing increase in intracellular calcium levels within nerve cells in response to ES (Al-Majed et al., 2000; Geremia et al., 2007, 2010). The cAMP pathway, in turn, upregulates the expression of growth factors BDNF, GDNF, and NGF, and enhances regeneration by upregulating the expression of neurotrophins and cytoskeletal proteins, such as GAP-43, tubulin, and actin (Willand et al., 2016; Ni et al., 2023). Additionally, ES activates the PI3K/Akt pathway in nerve cells by downregulating the expression of the growth inhibitor PTEN, which, in turn, upregulates the secretion of growth factors, such as BDNF, leading to enhanced axonal elongation (Singh et al., 2015).

### 4.2 Electrical stimulation and small extracellular vesicles

The physiological benefits of ES in PNR have been attributed to an increased secretion and uptake of sEVs (Hu et al., 2019; Debbi et al., 2022). Hu et al. (2019)demonstrated that ES at an intensity of 100 mV/mm for 1 h promotes the secretion of glutamate, an excitatory neurotransmitter in the nervous system, which is associated with an increase in sEV secretion in nerve cells. Glutamate binds to calcium-permeable ionotropic glutamate receptors on the surface of SCs, leading to a calcium influx. This sequential increase in the intracellular calcium levels stimulates the release of sEVs (Reddy et al., 2001; Savina et al., 2003). While research on the regenerative effects of ES has been gaining momentum, with numerous studies unveiling a stimulatory effect of ES on cells and sEV secretion (Brushart et al., 2002, 2005; Geremia et al., 2007; Koppes et al., 2014; Calvey et al., 2015; McLean and Verge, 2016; Hu et al., 2019; Fukuta et al., 2020; Roh et al., 2022; Li et al., 2023), the regulatory processes underlying these findings remain somewhat unexplored. Overall, ES enhances PNR across multiple domains, making it a noteworthy therapeutic approach.

## 5 Mechanotherapy and peripheral nerve regeneration

The implementation of non-invasive mechanotherapy has led to a significant enhancement in promoting PNR. Mechanotherapy, specifically through techniques, such as US and extracorporeal shock waves, facilitates cell regeneration by enhancing intercellular communication. The subsequent sections delve into the latest findings concerning the therapeutic utilization of US and extracorporeal shock waves for PNR, along with an exploration of the associated signaling pathways.

### 5.1 Ultrasound

US is known for its noninvasive and safe application in the medical field, serving both diagnostic and therapeutic purposes. US waves generate mechanical energy that stimulates tissue regeneration. Currently, the utilization of US spans a spectrum of intensities, including both high and low intensities, depending on its application. High-intensity US, characterized by an energy level exceeding 3 W/cm^2^, generates heat energy through molecular vibrations, mainly applied for localized tumor ablation in the treatment of prostate, liver, breast, and kidney cancers (Wu et al., 2004; Klingler et al., 2008; Crouzet et al., 2014; Peek and Wu, 2018). In contrast, low-intensity US, operating at energy levels below 1 W/cm^2^, does not generate thermal energy-associated tissue damage, making it more suitable for tissue regeneration (Acheta et al., 2021). With a depth of penetration of 3–5 cm at a frequency of 1 MHz and 1–2.5 cm at a frequency of 3 MHz, low-intensity US offers valuable potential for therapeutic applications (Draper et al., 1995; Hayes et al., 2004). They can be delivered in two different waveforms: continuous and pulsed. Among these, low-intensity pulsed ultrasound (LIPUS) is considered safer compared to high-intensity pulsed ultrasound, because it involves the delivery of low-intensity mechanical waves in a pulsatile manner, minimizing heat generation within the tissue while still promoting regeneration (Acheta et al., 2021; Grogan and Mount, 2023).

#### 5.1.1 Ultrasound stimulation to regulate intracellular signaling mechanisms

LIPUS-treated nerve cells exhibit a substantial increase in neurite outgrowth through the activation of the Netrin-1/DCC signaling pathway (Weng et al., 2018; Acheta et al., 2021). Netrin-1, a crucial guidance factor, plays a key role in neuronal development and contributes to peripheral nerve regrowth after injury (Dun and Parkinson, 2017). Despite the observed increase in Netrin-1 due to LIPUS, the precise molecular mechanism underlying the promotion of axonal elongation remains unclear (Yue et al., 2016; Ito et al., 2020; Acheta et al., 2021). LIPUS treatment also activates mechanoresponsive receptors in SCs and fibroblasts, leading to the activation of the PI3K/AKT signaling pathway. The mechanical force induces fibroblasts to enhance collagen production, thereby providing structural support for axonal repair (Bohari et al., 2012; Yue et al., 2016; Acheta et al., 2021; Hormozi-Moghaddam et al., 2021). Similarly, LIPUS-treated SCs show enhanced proliferation and expression of neurotrophic factors, especially GDNF, BDNF, and NGF, which drive axonal outgrowth. LIPUS enhances the redifferentiation of SCs into a myelinating state by upregulating the expression of myelinating factors, such as ERG2, NRG1, and MBP (Yue et al., 2016; Peng et al., 2020; Acheta et al., 2021). Notably, LIPUS-treated SCs exhibit increased expression of Cyclin D1, a protein that regulates cell proliferation. Elevated Cyclin D1 levels are associated with the inhibition of the Wnt/β-catenin signaling pathway, which in turn promotes nerve remyelination (Makoukji et al., 2012; Ren et al., 2018; Weng et al., 2018; Ito et al., 2020). Interestingly, LIPUS has been found to decrease the pro-inflammatory response of SCs by inhibiting inflammatory markers such as TNFα and IL-6. The pro-inflammatory response of SCs occurs during the early Wallerian degeneration phase of regeneration to regulate cellular debris clearance; however, if prolonged, it can impede axonal elongation. This finding suggests the potential application of US as an early intervention mechanism to accelerate axonal regeneration (Ito et al., 2020; Acheta et al., 2021).

#### 5.1.2 Low-intensity pulsed ultrasound stimulation and small extracellular vesicles

Intercellular communication through sEVs is another domain through which LIPUS can influence PNR. As previously discussed, sEVs play a crucial role in the regenerative cascade, relating their increased secretion to the regenerative outcome. Recently, Zeng et al. (2019) demonstrated that the treatment of lung cancer cells with US intensities ranging from 0.6 to 3.4 W/cm^2^ promoted sEV secretion. Furthermore, LIPUS stimulation of SCs led to the upregulation of the expression of myelin-related miRNAs, such as let-7c-5p and miR-34a-5p, in SC-derived sEVs. This variation in the miRNA profile of sEVs enhances their efficacy in PNR (Ye et al., 2023). sEVs containing elevated levels of let-7c-5p miRNA have been shown to induce increased NGF expression in nerve cells, subsequently activating the PI3K/AKT signaling pathway, leading to increased axonal outgrowth (Li et al., 2015; Ye et al., 2023). Although LIPUS enhances sEVs secretion and alters miRNA expression, the molecular mechanisms underlying these effects are poorly understood.

### 5.2 Extracorporeal shock wave

An extracorporeal shock wave (ESW) is an acoustic mechanical stimulation similar to an US wave, but it applies approximately 1,000 times the mechanical pressure (Romeo et al., 2014; Guo et al., 2022). ESW propagate a mechanical stimulus in the treated tissue, offering a therapeutic application for various conditions, including peripheral nerve injury. There are two types of ESW generators: focused and radial (Zwerver et al., 2016; Guo et al., 2022). A focused ESW (fESW) is commonly used for deep treatment areas, reaching depths of up to 12 cm from the surface (Dymarek et al., 2020). In contrast, a radially defocused waveform propagates through the tissue in spherical waves, reaching a depth of only 3–4 cm (Dymarek et al., 2020). A defocused waveform is more applicable for superficial treatments such as tibial bone fractures (Kertzman et al., 2017). Although focused waveforms are used for some PNR applications (Vahdatpour et al., 2016; Guo et al., 2022), defocused waveforms are considered more suitable for augmenting PNR (Hausner and Nogradi, 2013; d’Agostino et al., 2015; Zwerver et al., 2016). Regardless of its wave shape and form, ESWT generates a mechanical stimulus that provokes two physical effects: mechanotransduction and cavitation.

Mechanotransduction plays a significant role in PNR because the mechanical stimuli exerted by the surrounding regenerative microenvironment affect myelin gene regulation, SC differentiation, and axonal regeneration (Ingber, 2006). ESWT induces a mechanotransduction response through applied shear and pressure forces that impact cell membrane polarization, differentiation, proliferation, and intracellular regulatory processes (d’Agostino et al., 2015; Moya et al., 2018; Guo et al., 2022). For instance, the Piezo1 and Piezo2 channels, which are abundant in SCs, exhibit enhanced activation following ESW stimulation (Guo et al., 2022). In contrast, cavitation refers to the rapid implosion of air bubbles formed as a result of the negative pressure associated with ESWT. It generates a tensile force within the extracellular space that induces an indirect mechanical force on the surrounding cellular membrane, thereby promoting the secretion of intracellular growth factors (Lopez-Marin et al., 2018; Guo et al., 2022). ESWT often requires both mechanotransduction and cavitation to effectively regulate PNR.

#### 5.2.1 Extracorporeal shock wave therapy to regulate intracellular signaling mechanisms

Low-intensity ESWT (Li-ESWT) is associated with enhanced nerve regeneration, specifically, axonal outgrowth. Murata et al. demonstrated that *in vivo* Li-ESWT treatment involving 2,000 pulses at 0.08 mJ/mm^2^ and 4 Hz stimulated the expression of transcription factor ATF3 and enhanced axonal outgrowth in injured rat models (Murata et al., 2006; Wang et al., 2017). However, this treatment also caused injury to the sensory nerve fibers, indicating a potentially harmful effect of Li-ESWT. Alternatively, Li ESWT treatment using 300 pulses at an energy density of 0.06 mJ/mm^2^ and 3 Hz in rat models enhanced BDNF expression without causing nerve damage (Wang et al., 2017). Additionally, Hausner et al. (2012) reported improved directional specificity for axonal growth and enhanced nerve conduction velocity in rat models treated with 300 pulses of 0.1 mJ/mm^2^ Li-ESWT at 3 Hz. The substantial disparity in Li-ESWT stimulation parameters reflects the lack of a standardized approach for its application in PNR.

Treatment of neural stem cells with Li-ESWT has been shown to increase the activity of the PI3K/AKT and Wnt/β-catenin signaling pathways, both of which regulate axonal outgrowth (Xu et al., 2012; Weihs et al., 2014; Zhang J. et al., 2017; Lopez-Marin et al., 2018). Additionally, Wang et al. (2017) reported an increased activation of the PERK/ATF4 signaling pathway in DRG neurons following Li-ESWT treatment. Enhanced axonal elongation following Li-ESWT treatment is primarily attributed to the upregulation of the growth factor BDNF via the PERK/ATF4 and PI3K/AKT signaling pathways. In SCs, Li-ESWT activates the phosphorylation of PERK and the translation of the ATF4 gene, which is associated with growth factor overexpression (Schuh et al., 2016; Wang et al., 2017). Li-ESWT is also believed to mediate macrophage inflammatory responses during PNR. Stimulation of macrophage cell cultures with Li-ESWT involving 400 pulses and energy density of 0.03–0.1 mJ/mm^2^ at a frequency of 3.5 Hz promoted the expression of the anti-inflammatory cytokine IL-10 and M2 marker genes ALOX15, MRC1, and CCL18, associated with accelerated axonal outgrowth (Sukubo et al., 2015). In addition, Li-ESWT-treated macrophages showed reduced expression of the inflammatory marker IL-1β (Sukubo et al., 2015). These effects highlight the potential of Li-ESWT to regulate inflammatory responses during Wallerian degeneration, thereby expediting axonal elongation and nerve regeneration (Sukubo et al., 2015; Guo et al., 2022).

#### 5.2.2 Extracorporeal shock wave therapy and small extracellular vesicles

The effect of ESWT on sEV release to facilitate PNR remains relatively underexplored. Gollmann-Tepeköylü et al. examined the effect of ESWT on sEV release for ischemic heart disease improvement and reported that the mechanical stimulation resulting from ESWT drives an increase in sEV release (Gollmann-Tepekoylu et al., 2020). Although this study did not investigate the release of sEVs in the context of the PNS, it does highlight a probable correlation between Li-ESWT mechanical stimulation and sEV secretion. Moreover, ESWT has been found to modulate the Wnt/β-catenin pathway, a potential regulatory pathway for sEV-mediated intercellular communication. Further research is needed to explore the impact of Li-ESWT on sEV release as component of the peripheral nerve regenerative cascade.

### 5.3 A systematic comparison between mechanotherapy platforms

Considering the observed benefits of both LIPUS and ESWT, their application *in vivo* results in enhanced functional recovery following sciatic nerve injury. This improvement is attributed to accelerated axonal regeneration and early target-organ reinnervation. More specifically, US treatment is associated with increased nerve fiber density, larger axons, thicker myelin sheaths, and faster nerve conduction velocities, all of which offer expedited regeneration (Ito et al., 2020). In addition, US accelerates Wallerian degeneration, increases SCs proliferation, and promotes the secretion of the neurotrophic factor CNTF and growth factors BDNF and NGF (Daeschler et al., 2018; Ito et al., 2020). In contrast, ESWT contributes mainly to the secretion of the growth factor BDNF (Weihs et al., 2014; Schuh et al., 2016). Compared with US, ESWT elicits a more rapid inflammatory response, assisting in the initial phase of Wallerian degeneration (Daeschler et al., 2018; Ito et al., 2020). *In vitro* investigations of ESWT revealed increased proliferation of SCs, along with enhanced expression of regenerative markers GFAP and c-Jun (Wang et al., 2017; Daeschler et al., 2018). However, ESWT has been reported to be most effective in the Wallerian degeneration stage, with limited significant improvements thereafter (Hausner et al., 2012). Both therapeutic modalities share similarities in application and outcomes. However, a significant gap remains in understanding the molecular mechanisms governing both approaches in PNR.

## 6 Future outlook

Our comprehension of PNR has advanced steadily over time, yet significant prospects for improvement remain. The timeline of regeneration, especially concerning the cellular inflammatory responses during Wallerian degeneration, remains ambiguous. While the proinflammatory stage during PNR is crucial for facilitating axonal outgrowth, prolonged inflammation can impede regeneration. The overlapping stages of regeneration create a gap in our understanding of cellular communication within the regenerative cascade. With growing research on sEVs and their role in PNR, they hold promise as a tool for studying intercellular communication. However, further insight is needed into the uptake mechanisms of sEVs in the PNS.

A thorough characterization of sEV cargo profiles, including miRNAs and proteins, across all responder cells during regeneration, is essential. Proteomic and transcriptomic analysis can be utilized to identify and characterize the sEVs with regard to their cellular origin, as recent studies revealed SC-and Fibroblast-derived sEV differentially expressed genes (Wei et al., 2019; Zhou et al., 2024). However, more research is needed to fully understand the differential characterization of peripheral and central nerve-derived sEVs, and their effect on PNR. Another limitation is the comparative analysis of sEV characterization between *in vivo* and *in vitro* models, as a deeper understanding of the disparities between *in vivo* and *in vitro* multi-cell culture models for sEV isolation is required. In addition, further research is required to characterize the distinct sEV cargo profiles associated with different SC phenotypes in the regenerative cascade. Moreover, there exists a limitation in replicating PNR in the realistic 3D biomimetic models, as it integrates a multi-cell culture to investigate sEV communication. Hence, mapping sEVs involvement in cellular communication at each stage of the regeneration cascade requires further investigation. Recent findings have proposed another EV subgroup, matrix-bound vesicles (MBVs), tailored specifically to the ECM. Understanding the influence of MBVs on the regenerative cascade holds promise, addressing gaps in knowledge pertaining to ECM cellular communication. Similarly, it is necessary to understand the niches of all EV subgroups in the pathophysiology and treatment of peripheral nerve injury.

Future research into ES and mechanotherapy holds potential to advance clinical strategies for promoting PNR. However, an in-depth understanding of the molecular mechanisms underlying these modalities needs to be improved. The diverse parameters for US and ESWT stimulations, both *in vivo* and *in vitro*, present challenges in assessing their specific benefits in PNR, necessitating standardized protocols. As ESWT gains attraction in this field, a clear molecular understanding of its regenerative effects becomes necessary.

While ES offers notable benefits in PNR, our comprehension of the underlying molecular mechanisms is limited. To gain a deeper understanding, it is crucial to examine the effects of ES on the biogenesis and secretion of sEVs within the regenerative cascade. Furthermore, exploring the intersection of electrical and mechanical therapeutic stimulations and sEV communication in PNR also holds paramount importance. Applications such as biocompatible conductive hydrogels and piezoelectric nanofibers in an *in vivo* PNI model can help us study how mechanical and electrical stimulation affect sEV communication during PNR. This in turn can help us address the challenges associated with PNI and revolutionize regenerative treatment modalities.

## Author contributions

YI: Investigation, Visualization, Writing – original draft, Writing – review & editing. LE: Conceptualization, Supervision, Writing – review & editing.
